# Dyslipidemia in adolescents and young adults with type 1 and type 2 diabetes: a retrospective analysis

**DOI:** 10.1186/s13633-020-00081-7

**Published:** 2020-06-11

**Authors:** Grace Kim, Daniel DeSalvo, Danielle Guffey, Charles G. Minard, Constance Cephus, Douglas Moodie, Sarah Lyons

**Affiliations:** 1 Department of Pediatrics, Section of Diabetes and Endocrinology, Texas Children’s Hospital, Baylor College of Medicine, Houston, TX 77030 USA; 2 Dan L. Duncan Institute for Clinical and Translational Research, Baylor College of Medicine, Houston, TX 77030 USA; 3 Department of Pediatrics, Section of Cardiology, Texas Children’s Hospital, Baylor College of Medicine, Houston, TX 77030 USA

**Keywords:** Dyslipidemia, Type 1 diabetes, Type 2 diabetes, LDL cholesterol, HDL cholesterol

## Abstract

**Background:**

Youth onset type 1 diabetes (T1D) and type 2 diabetes (T2D) is increasing and associated with earlier vascular complications and mortality. Dyslipidemia is an important modifiable cardiovascular (CVD) risk factor that is under-recognized and undertreated in youth with T1D and T2D. Given this, we evaluated the prevalence and associations between lipid concentrations and clinical CVD risk factors in youth with T1D compared to T2D at our large ethnically diverse diabetes center.

**Methods:**

A retrospective chart review was performed, evaluating patients with T1D or T2D seen at least once in clinic from 2015 to 2017, age 10–22 years of age, duration of diabetes at least 6 months on the date of most recent LDL-cholesterol (LDL-C) concentration, and not on statin therapy. We performed independent and multivariable linear regressions of LDL-C and HDL-cholesterol (HDL-C) concentrations.

**Results:**

There were 32.7% with T1D (*n* = 1701) and 47.7% with T2D (*n* = 298) with LDL-C above recommend goal (> 100 mg/dL/2.6 mmol/L). Furthermore, there were 9% with T1D and 16.4% with T2D with LDL > 130 mg/dL (> 3.4 mmol/L), who likely met criteria for starting statin therapy. Higher LDL-C and/or lower HDL-C were associated with increased age, diabetes duration, higher HbA1C, female sex, Hispanic ethnicity, obesity, and T2D. After adjusting for these risk factors in a multivariable linear regression model, the association of higher LDL-C and lower HDL-C was higher with T2D than T1D.

**Conclusions:**

This highlights the need for more aggressive dyslipidemia screening and treatment in youth with diabetes, especially T2D. At our institution we have created and instituted quality improvement algorithms to try to address this need.

## Background

Cardiovascular disease (CVD) is a major cause of morbidity and mortality in those with type 1 and type 2 diabetes [1–3]. Youth onset type 1 diabetes (T1D) and type 2 diabetes (T2D) is increasing in prevalence and associated with early atherosclerosis, CVD risk factors, vascular complications, and mortality [4–9]. Youth onset T2D compared to youth onset T1D is associated with earlier diabetes related complications, increased CVD risk factors, and increased mortality [10–13]. These underline the importance of screening for and treating CVD risk factors in all youth with both T1D and T2D.

Dyslipidemia is an important modifiable CVD risk factor. Evidence-based recommendations from The International Society for Pediatric and Adolescent Diabetes (ISPAD) and American Diabetes Association (ADA) recommend goal low density lipoprotein cholesterol (LDL-C) of < 100 mg/dL (2.6 mmol/L) in youth with diabetes [14, 15]. For pediatric patients above age 10 years of age with elevated LDL-C despite medical nutrition therapy and lifestyle intervention, the guidelines recommend consideration of statin pharmacotherapy based on the severity of LDL-C concentrations and presence of cardiovascular risk factors. However, dyslipidemia is underrecognized and undertreated in youth with diabetes, as a substantial proportion of youth are not meeting recommended guidelines [16–18].

As incidence of youth onset T1D and T2D have been increasing across many ethnic minorities, it is important to look at trends among the ethnically diverse populations [19, 20]. Given this, we evaluated the prevalence of dyslipidemia in youth with diabetes at our large ethnically diverse pediatric diabetes care center with a high Hispanic population. We also evaluated for associations between lipid concentrations and CVD risk factors in those with T1D and T2D, and we analyzed for differences in presence of modifiable CVD risk factors between T1D and T2D with LDL-C not at ADA/ISPAD goal (< 100 mg/dL/< 2.6 mmol/L). Our hypotheses were: 1) a majority of the population would have elevated LDL-C concentrations not at goal, 2) higher LDL-C concentrations and lower HDL-C concentrations would be associated with T2D than T1D after adjusting for risk factors, 3) those with LDL-C not at goal, that T2D would be associated with more modifiable cardiovascular risk factors than T1D.

## Methods

### Protocol and measurements

Baylor College of Medicine’s Institutional Review Board approved this retrospective chart review. Patient characteristics and clinical and laboratory data were extracted from Texas Children’s Hospital’s (TCH) electronic medical records (EMR) using Epic Population Health Registry. Variables included age, sex, race/ethnicity, last hemoglobin A1C (HbA1C), body mass index (BMI), diabetes duration, insulin modality [pump therapy, multiple daily injections (insulin to carbohydrate ratio and correction factor), fixed doses, other], hypertension, and tobacco use. We were unable to assess exercise and family history of CVD due to limitations in the electronic medical records. TCH Diabetes Population Health Registry is an institutional based registry that collects discrete data from clinical documentation, laboratory reporting, and patient self-reporting. BMI was calculated in Epic using CDC charts from 2000 for age and sex [21], and categorized according to pediatric standards of obese (BMI > 95th%tile), overweight (BMI 85 to <95th%tile) and normal (BMI <85th%tile) [22]. Lipid panel was ordered at provider discretion based on established guidelines, but without a standardized process or protocol in place from TCH Laboratories or Quest Diagnostic Laboratories. Clinic HbA1C was obtained from DCA Vantage™ Analyzer, Seimens.

### Population/subjects

Inclusion criteria were a diagnosis of T1D or T2D seen in clinic at least once at TCH Diabetes Care Center in Houston, Texas, USA, from December 1, 2015-December 1, 2017 with age 10–22 years of age and duration of diabetes at least 6 months on the date of most recent LDL-C concentration. Individuals with LDL-C concentrations that could not be calculated or directly measured by the lab or not screened, or with prescription for statin therapy at time of most recent LDL-C concentration were excluded.

### Data analysis

Patient and clinical characteristics were summarized using mean with standard deviation, median with 25th and 75th percentiles, and frequency with percentage. The summary statistics were stratified by diabetes type and compared using two sample t-test, Wilcoxon rank sum test, Fisher’s exact test, or Chi-square test.

We analyzed lipid concentrations both continuously and categorically based on recommendations by National Heart Lung Blood Institute Expert Panel [23]. LDL-C was divided into LDL-C < 100 mg/dL (< 2.6 mmol/L), 100–129 mg/dL (2.6–3.3 mmol/L), 130–159 mg/dL (3.4–4.1 mmol/L), and > 160 mg/dL (> 4.1 mmol/L); high density lipoprotein cholesterol (HDL-C) was categorized as HDL-C < 40 mg/dL (< 1 mmol/L) and HDL-C > 40 mg/dL (> 1 mmol/L); total cholesterol (TC) was categorized as TC < 200 mg/dL (< 5.2 mmol/L) and TC > 200 mg/dL (> 5.2 mmol/L); triglycerides (TG) were classified as TG < 130 mg/dL (< 1.5 mmol/L) and TG > 130 mg/dL (> 1.5 mmol/L).

We assessed independent (unadjusted) linear associations of LDL-C and HDL-C concentrations with clinical and CVD risk factors of age, sex, race/ethnicity, BMI percentile, diabetes duration, HbA1C, and diabetes type. Significant risk factors were then combined in a multivariable (adjusted) linear model. We also evaluated in a multivariable logistic regression model for LDL-C > 100 mg/dL (> 2.6 mmol/L) and HDL-C < 40 mg/dL (< 1 mmol/L). Furthermore, in those with LDL-C not at ADA/ISPAD goal (< 100 mg/dL/2.6 mmol/L), we evaluated the prevalence of modifiable CVD risk factors. Differences were considered statistically significant if *p* < 0.05. Statistical analyses performed using Stata v 15.1 (StataCorp, College Station, TX, USA).

## Results

Of the 2077 patients that met inclusion criteria, 44 patients were excluded due to having lipid measurements unable to be calculated or not screened and 34 (including 22 with T1D and 12 with T2D) who had current statin prescriptions at time of lipid concentration. Of the 1999 remaining patients, 1701 (85%) patients had T1D and 298 (15%) had T2D (Table 1). There was a difference in race and ethnicity between those with T2D and T1D (*p* < 0.001), with a higher percentage of Hispanic White and Non-Hispanic Black in T2D (T2D vs T1D; 57.8% vs 22.9, 29.3% vs 17.2% respectively) and higher percentage of Non-Hispanic White in T1D (T1D vs T2D; 54.2% vs 6.8%). There was also a difference in BMI between T2D and T1D (*p* < 0.001); T2D had higher rate of obesity (T2D vs T1D; 80.1% vs 16.5%), whereas T1D had higher rate of overweight BMI (T1D vs T2D; 22.7% vs 13.9%). In both groups, there were a high percentage of patients who never used tobacco (T1D vs T2D; 70.9% vs 78.5%) but also a high percentage of patients who were not asked (T1D vs T2D; 27.7 vs 19.5%).

**Table 1:** Clinical Characteristics in T1D and T2D

	T1D	T2D	p-value
	N total = 1701	N total = 298	
**Age, yrs** (mean + SD)	15.4 + 2.8	16.2 + 2.1	< 0.001
**HbA1C**, % (mean + SD)	9 + 1.9	8.4 + 2.7	< 0.001
**Duration diabetes**, years (median, IQR)	6 (3.2, 9.1)	2.5 (1.5, 4.7)	< 0.001
**Sex, N** N (%)	N = 1701	N = 298	< 0.001
Female	875 (51.4)	196 (65.8)	
Male	826 (48.6)	102 (34.2)	
**Race/Ethnicity, N** N (%)	*N* = 1667	*N* = 294	< 0.001
Hispanic White	382 (22.9)	170 (57.8)	
Non-Hispanic White	904 (54.2)	20 (6.8)	
Hispanic Black	6 (0.4)	3 (1.0)	
Non-Hispanic Black	286 (17.2)	86 (29.3)	
Hispanic other	19 (1.1)	8 (2.7)	
Non-Hispanic other	70 (4.2)	7 (2.4)	
**BMI, N** N (%)	*N* = 1687	*N* = 296	< 0.001
Normal (BMI < 85%)	1025 (60.8)	18 (6.1)	
Overweight (BMI 85–94%)	383 (22.7)	41 (13.9)	
Obese (BMI > 95%)	279 (16.5)	237 (80.1)	
**Insulin modality, N** N (%)	*N* = 1700	N = 298	< 0.001
Multiple daily injections	723 (42.5)	9 (3.0)	
Pump	720 (42.4)	1 (0.3)	
Fixed Dose	254 (14.9)	149 (50.0)	
Other	3 (0.2)	139 (46.6)	
**Tobacco Use, N** N (%)	N = 1701	N = 298	0.003
Yes (past or present)	25 (1.4)	6 (2)	
Never	1205 (70.9)	234 (78.5)	
Not Asked	471 (27.7)	58 (19.5)	

p-values for mean comparisons performed with t-test

*p*-values for median comparisons using Two-sample Wilcoxon rank-sum (Mann-Whitney) test

*p*-values calculated with exact testing for categorical variables when possible otherwise chi-square test

Summary statistics for cholesterol concentrations are summarized in Table 2. T2D had higher median LDL-C, lower median HDL-C, higher median TC, and higher median TG. There were 556/1701 (32.7%) with T1D and 142/298 (47.7%) with T2D not at ADA/ISPAD LDL-C goal (< 100 mg/dL or 2.6 mmol/L). Of those with LDL-C above ADA/ISPAD goal, 153/1701 (9%) with T1D and 49/298 (16.4%) with T2D had LDL-C > 130 mg/dl (> 3.4 mmol/L).

**Table 2:** Lipid Concentrations in T1D and T2D

	T1D	T2D	p-value
	N total = 1701 N (%)	N total = 298 N (%)	
**LDL-C (mg/dL)**			< 0.001
< 100	1145 (67.3)	156 (52.3)	
100–129	403 (23.7)	93 (31.2)	
130–159	112 (6.6)	38 (12.8)	
> =160	41 (2.4)	11 (3.7)	
Median	87 mg/dL = 2.3 mmol/L	97.5 mg/dL = 2.5 mmol/L	< 0.001
**HDL-C (mg/dL)**			< 0.001
> =40	1532 (90.1)	160 (53.7)	
< 40	169 (9.9)	138 (46.3)	
Median	55 mg/dL = 1.4 mmol/L	41 mg/dL = 1.1 mmol/L	< 0.001
**Total Cholesterol (mg/dL)**			0.250
< 200	1406 (82.7)	238 (79.9)	
> =200	295 (17.3)	60 (20.1)	
Median	165 mg/dL = 4.3 mmol/L	169.5 mg/dL = 4.4 mmol/L	0.026
**Triglycerides (mg/dL)**			< 0.001
< 130	1305 (76.7)	137 (46.0)	
> =130	396 (23.3)	161 (54.0)	
Median	86 mg/dL = 1.0 mmol/L	140.5 mg/dL = 1.6 mmol/L	< 0.001

p-values calculated with exact testing for categorical variables when possible otherwise chi-square test

Higher LDL-C was independently associated with increased age, diabetes duration, and HbA1C, Hispanic ethnicity, female sex, overweight and obesity, and T2D than T1D (Table 3). After adjusting for age, sex, race/ethnicity, BMI, diabetes duration, and HbA1C in a multivariable analysis, the association of increased LDL-C was higher with T2D than T1D (*p* = 0.005) (Table 4).

**Table 3:** Independent (Unadjusted) Linear Regression for LDL-C

	Coefficient	95% Confidence Interval		p-value
Age	1.26	0.81	1.71	< 0.001
Race/Ethnicity				< 0.001
Hispanic White	Reference			
Non-Hispanic White	−5.57	−8.56	−2.59	< 0.001
Hispanic Black	17.86	−0.78	36.5	0.060
Non-Hispanic Black	1.05	−2.67	4.78	0.578
Hispanic Other	−2.21	−13.14	8.72	0.692
Non-Hispanic Other	−0.35	−7.10	6.40	0.919
Male	−7.04	−9.52	−4.56	< 0.001
BMI				< 0.001
Normal	Reference			
Overweight	7.35	4.19	10.52	< 0.001
Obese	10.13	7.18	13.09	< 0.001
Diabetes Duration	0.51	0.20	0.82	< 0.001
Hgb A1c	2.87	2.28	3.46	< 0.001
Type 2 Diabetes	9.12	5.65	12.60	< 0.001

**Table 4:** Multivariable (Adjusted) Linear Regression for LDL-C

	Coefficient	95% Confidence Interval		p-value
Type 2 diabetes	6.19	1.90	10.48	0.005
Age	0.82	0.34	1.30	0.001
Male	−6.10	−8.55	−3.66	< 0.001
Race/Ethnicity				0.302
Hispanic White	Reference			
Non-Hispanic White	−0.64	−3.74	−2.45	0.684
Hispanic Black	16.83	−0.96	34.61	0.064
Non-Hispanic Black	−1.00	−4.60	2.60	0.586
Hispanic Other	1.12	−9.40	11.64	0.835
Non-Hispanic Other	3.99	−2.58	10.56	0.234
BMI				< 0.001
Normal weight	Reference			
Overweight	5.18	2.03	8.32	0.001
Obese	7.05	3.65	10.46	< 0.001
Diabetes Duration	0.46	0.11	0.80	0.010
Hgb A1c	2.97	2.36	3.57	< 0.001

Lower HDL-C was independently associated with increased age, Hispanic ethnicity, female sex, overweight and obesity, and T2D than T1D (Table 5). Increased diabetes duration and HbA1C were each associated with increased HDL-C. After adjusting for age, sex, race/ethnicity, BMI, diabetes duration, and HbA1C in a multivariable analysis, the average HDL-C was lower among T2D than T1D (*p* < 0.001) (Table 6).

**Table 5:** Independent (Unadjusted) Linear Regression for HDL-C

	Coefficient	95% Confidence Interval		p-value
Age	−0.91	−1.15	−0.67	< 0.001
Race/Ethnicity				< 0.001
Hispanic White	Reference			
Non-Hispanic White	3.83	2.24	5.41	< 0.001
Hispanic Black	5.83	−4.06	15.73	0.248
Non-Hispanic Black	5.18	3.21	7.16	< 0.001
Hispanic Other	−3.94	−9.75	1.86	0.183
Non-Hispanic Other	5.75	2.17	9.33	0.002
Male	−2.90	−4.22	−1.58	< 0.001
BMI				< 0.001
Normal	Reference			
Overweight	−3.61	−5.22	−2.01	< 0.001
Obese	−12.31	−13.81	− 10.81	< 0.001
Diabetes Duration	0.28	0.11	0.45	< 0.001
Hgb A1c	0.52	0.20	0.84	< 0.001
Type 2 Diabetes	−14.72	−16.46	−12.97	< 0.001

**Table 6:** Multivariable (Adjusted) Linear Regression for HDL-C

	Coefficient	95% Confidence Interval		p-value
Type 2 diabetes	− 10.20	− 12.34	−8.06	< 0.001
Age	−0.79	−1.03	−0.55	< 0.001
Male	−4.55	−5.77	−3.33	< 0.001
Race/Ethnicity				< 0.001
Hispanic White	Reference			
Non-Hispanic White	−1.39	−2.93	0.16	0.078
Hispanic Black	6.66	−2.21	15.52	0.141
Non-Hispanic Black	3.88	2.08	5.67	< 0.001
Hispanic Other	−5.13	−10.38	0.11	0.055
Non-Hispanic Other	0.28	−3.00	3.55	0.869
BMI				< 0.001
Normal weight	Reference			
Overweight	−3.58	−5.14	−2.01	< 0.001
Obese	−9.05	−10.75	−7.35	< 0.001
Diabetes Duration	0.05	−0.12	0.22	0.582
Hgb A1c	0.14	−0.16	0.44	0.366

In individuals with LDL-C not at ADA/ISPAD goal, those with T2D had higher rate of obesity (T2D vs T1D; 79.3% vs 21.5%, p < 0.001) and HDL-C < 40 mg/dL (< 1 mmol/L) (T2D vs T1D; 47.9% vs 10.6%, p < 0.001) compared to T1D.

## Discussion

As incidence of youth onset T1D and T2D is increasing and associated with earlier vascular complications and mortality, it is important to analyze dyslipidemia prevalence and associations with CVD risk factors between T2D and T1D in the diverse pediatric population. Our study represents retrospective data from a single large diabetes care center with an ethnically diverse population, which showed a high percentage of youth with LDL-C not at goal (< 100 mg/dL/ < 2.6 mmol/L): 32.7% (556/1701) with T1D and 47.7% (142/298) with T2D. Of those that likely met criteria to start statin therapy (LDL > 130 mg/dL/3.4 mmol/L), there were 9% (153/1701) with T1D and 16.4% (49/298) with T2D. These numbers slightly underestimate the total population with dyslipidemia at our institution as we excluded those that were already started on statin treatment, and for those whom LDL-C could not be calculated due to elevated TG. However, this prevalence of dyslipidemia in our patient population is lower than we hypothesized but overall comparable to, if not slightly less, than other population studies. In the SEARCH for Diabetes in Youth population, a national multicenter study in the United States evaluating children and young adults with diabetes, it was reported in 2006 that 47% of patients with T1D above age 10 years had LDL > 100 mg/dL (> 2.6 mmol/L), with 15% > 130 mg/dL (> 3.4 mmol/L), and 57% of those with T2D had LDL > 100 mg/dL (> 2.6 mmol/L), with 24% with LDL > 130 mg/dL (> 3.4 mmol/L) [24]. Since then other studies worldwide have shown varying high prevalence of dyslipidemia in the pediatric diabetes population. In 2008, a single center in the UK showed that 38% of children with T1D had LDL > 100 mg/dL (> 2.6 mmol/L), and 10.8% with LDL > 130 mg/dL (> 3.4 mmol/L) [25]. More recently, in 2015 another study at a center in Brazil, showed high LDL > 100 mg/dL in 44% of T1D (age 5–31 years of age), and a study from a center in Turkey in 2017 showed 21% with high LDL > 130 mg/dL (> 3.4 mmol/l) [26]. For type 2 diabetes in youth, a single center in California, USA in 2008, showed that 39.4% of youth with T2D had LDL > 130 mg/dL (> 3.4 mmol/L). In 2017, a study from India showed prevalence of high LDL-C > 100 mg/dL (> 2.6 mmol/L) was 64.5% in youth with T2D [27]. One possibility of our prevalence of dyslipidemia being slightly lower than expected, is the increased attention lately to the high rates of dyslipidemia in pediatric patients with diabetes. Also our screening rate was surprisingly high, > 98%, which leads to early detection and hopefully early discussions about dyslipidemia management. Despite these, the prevalence of dyslipidemia in our diabetes population is still high, which shows the need for improved management of dyslipidemia in pediatric patients with diabetes.

Our study population is an ethnically diverse population with high Hispanic prevalence (57.8% of T2D and 22.9% of T1D), higher than that of the national multicenter SEARCH for Diabetes in Youth population (27.6% of T2D and 13.0% of the T1D), or of the NHANES (National Health and Nutrition Examination Survey) adolescent populations [12, 28]. Racial disparities have been reported in both T1D and T2D where minorities compared to Non-Hispanic White tend to have increased CVD risk factors including dyslipidemia, however the relationship and mechanism is unclear and complicated [7, 29, 30]. Our study population with a high Hispanic proportion, showed a high prevalence of dyslipidemia, emphasizing the importance of screening and treating dyslipidemia in these youth.

On further analysis of our study population, higher LDL-C and/or lower HDL-C were independently associated with older age, Hispanic ethnicity, female sex, overweight and obesity, longer diabetes duration, higher HbA1C, and T2D. We chose these risk factors to analyze as they have been associated with dyslipidemia [5, 7, 25, 31–36], and to evaluate if they were also significantly associated in our population. The role of sex in dyslipidemia in those with T1D or T2D is varied, where generally sex is not associated with increased CV risk factors in those with diabetes [7], though another study showed that females had higher LDL-C and higher TC [5], while another study showed that males were associated with higher odds of progression of HDL-C [31]. Higher BMI is associated with dyslipidemia in many studies and our study was concordant with this [32–34]. Worsening glycemic control has been associated independently with dyslipidemia in multiple studies [25, 31, 32, 34–36]. Higher duration of diabetes is also typically associated with worsening dyslipidemia [25]. It is interesting that in our study, worsening glycemic control and longer duration of diabetes were associated with increased HDL-C concentrations. One possibility for this association is lower HDL-C was associated with T2D, and T2D was associated with lower duration of diabetes and lower HbA1C in our population. We did try to evaluate these factors separately in T1D and T2D. In T1D, higher HbA1C was again associated with HDL-C, however duration of diabetes was not significant. In T2D, both factors were not significant, likely due to not enough of a sample size (data not shown).

We then adjusted for the significant risk factors, to evaluate whether higher LDL-C and lower HDL-C would be still be associated with specifically T2D versus T1D, as the prevalence of T2D is increasing in pediatrics, and known to be associated with earlier microvascular and macrovascular complication [10, 11]. In our population, after adjusting for age, sex, race/ethnicity, obesity, diabetes duration, and HbA1C, both higher LDL-C and lower HDL-C concentrations were associated T2D than T1D. We found that the type of diabetes, specifically T2D, is associated with higher LDL-C and lower HDL-C even after accounting for the major risk factors that contribute to CVD. Additionally, for patients with LDL-C not at goal, presence of other CVD risk factors of obesity and low HDL-C were associated with T2D than T1D. Our analysis is concordant with recent findings from the national multicenter SEARCH for Diabetes in Youth population, where the prevalence of > 2 CVD risk factors among youth with T2D was 8–10 times as high as youth with T1D [12]. These are consistent with studies showing youth onset T2D is associated with earlier complications than youth onset T1D or adults with T2D [9–11, 37]. It has been suggested that there are differences in the pathophysiology of dyslipidemia in T2D vs T1D, however this mechanism has not yet been elucidated [38]. Thus, we need to be vigilant with dyslipidemia management in youth onset diabetes, especially T2D.

At our institution, lipid screening was at the discretion of the individual providers, and not based on standardized institutional protocols for screening and ongoing management. This led to limitations of lipid concentrations that were both fasting and non-fasting, leading to a small percentage of LDL-C concentrations that were unable to be calculated due to high triglycerides. Another limitation is that there were high rates of unreported tobacco status. Other cardiovascular risk factors including family history of cardiovascular disease, hypertension, diet history, and exercise were other factors unable to be obtained due to limitations with the electronic health registry. In addition, our results may not be generalizable to the whole population as we present a retrospective chart review of our single tertiary diabetes care center with a high Hispanic population. Also, these findings do not necessarily predict CVD outcomes as we focused on the associations with lipid abnormalities and diabetes. Of note, the pediatric guidelines are extrapolated from adult data, thus in general more long term studies are needed to know the exact long term outcomes of improved dyslipidemia control in pediatric patients with diabetes. Despite the limitations, we had a large diverse population with T1D and T2D for analysis.

## Conclusions

In summary, in our large diabetes care center with an ethnically diverse population, our study showed a high percentage of pediatric patients with T1D and T2D with high LDL-C and low HDL-C concentrations that do not meet recommended LDL-C goals (< 100 mg/dL/ < 2.6 mmol/L): 32.7% in T1D and 47.7% in T2D. Those who likely met criteria to start statin therapy with LDL > 130 mg/dL (> 3.4 mmol/L), were 9% in T1D and 16.4% in T2D. We identified the association of high LDL-C and low HDL-C concentrations was higher with T2D than T1D when adjusting for CVD risk factors. Also, for those with LDL-C not at ADA/ISPAD goal, patients with T2D had more cardiovascular risk factors. These results are not entirely unexpected, but offer more insight into the population we treat at our diabetes care center and emphasize the continued need for rigorous screening and treatment for dyslipidemia in patients with T1D and especially T2D. Improved treatment may include statin therapy, which we noted low rates of statin use in both the T1D and T2D populations at our center. Possible reasons for low rates of statin use include provider comfort with starting statin therapy in pediatric patients, lack of knowledge of guidelines, concern of teratogenicity risk, or lack of acceptance by families.

Based on the knowledge we gained in this study, we initiated a quality improvement initiative at our institution. We are creating a dyslipidemia screening and management (including statin therapy) algorithm, based on evidence based guidelines and risk factors [14, 23]. To alert clinicians of the need to screen, we are creating EMR based alerts based on the patient’s last LDL-C concentrations and diabetes type. To better educate patients, we are standardizing dietitian education material for dyslipidemia. We will be monitoring if the percentage of LDL-C not at goal is improved based on these targeted quality improvement initiatives.

## Data Availability

The datasets used and/or analyzed during the current study are available from the corresponding author on reasonable request.

## Abbreviations

T1D: Type 1 diabetes mellitus
T2D: Type 2 diabetes mellitus
CVD: Cardiovascular disease
ISPAD: International Society of Pediatrics and Adolescent Diabetes
ADA: American Diabetes Association
LDL-C: LDL cholesterol
HDL-C: HDL cholesterol
TC: Total cholesterol
TG: Triglycerides
TCH: Texas Children’s Hospital
EMR: Electronic medical records
HbA1C: Hemoglobin A1C
BMI: Body mass index
